# The Study of Anatomy

**Published:** 1897-08

**Authors:** W. C. Barrett

**Affiliations:** Buffalo, N. Y.


ARTICLE V.

### THE STUDY OF ANATOMY.

BY W. C. BARRETT, M. D., D. D. S., M. D. S., BUFFALO, N. Y.

This association has wrought a great work in securing the adoption of something like uniformity of action in the admission of students, and in the raising of the general educational standard. If one would have some comprehension of its beneficent influence, he has but to reflect upon what was the general character of American schools, and what their reputation abroad before the organization of the National Association of Dental Faculties, as compared with the present condition. And yet it has done but a small proportion of its manifest duty. Its accomplishments have been elementary.

It is not too much to say that our professional reputation must be what our colleges make it. We are the educators of those who are to be the leaders in the professional matters of the future. The next generation of dentists will be what we shall make it. Legislators may pass laws to regulate and restrict dental practice, but the stream can rise no higher than the fountain-head, and the practitioner of to-morrow must get his training and derive his professional knowledge from the school to-day. He must enter the profession by submitting himself to our guidance. The colleges are the fountain-head, and the stream will be limpid or foul according to whether we purify or contaminate it.

This should be a proud position. It certainly is a responsible one, and woe betide the college professor who does not realize his accountability. The man who accepts the honor which may appertain to this distinguished station, without striving his utmost to be in every way worthy of it, to fulfill every duty with an eye single to the best interests of student and profession, is unworthy a place in our ranks. He who assumes to arm the young men of our county for the battle of life, to fit them and equip them for an honorable career simply that he may minister to his own good, who takes the teacher's place and ascends the instructor's rostrum from selfish motives, is a worse hypocrite than the preacher whose every-day life belies his own sermons.

I believe that we are all sincere in desiring to make our schools, and through them the profession, all that they should be. To secure this it is not enough that we look solely to the preliminary qualifications of those whom we accept as candidates for a confidential position in American families. We need to make our instruction as perfect as possible. This cannot be done unless there is a generally accepted standard, and some uniformity in system. At present one of our greatest sources of weakness lies in the fact that there is no common comprehension of a standard of methods. One school begins instructions with the alphabet, proceeds to the construction of simple words, and by regular gradations to the building up of sentences. Another commences by an analysis of the sentence into its component words, and then studies the elementary symbols constituting the words.

That is, one teacher is synthetical, and the other strictly analytical. A student takes his first and second year in one school, and then circumstances or inclination cause him to finish his course at another. He commences under analytical teachers, and closes with a school that only arrives at the stage of analysis in the closing year. Hence, in reality that student never reaches the end of any regularly graded course. In this way the practical efficiency of that graduate can never be assured. Let me illustrate this by the various methods of arriving at a knowledge of that nasal study in all schools that attempt to teach the healing art—anatomy.

Some teachers open their course with an examination of the elements of which the human body is composed. That is, they begin with histology. They commence with the cell, and after having given a fair knowledge of that, they proceed to construct the cells into tissues, which are then considered. Then the tissues are built into organs, and finally the organs into the systems which they compose, and they do not arrive at a consideration of the human body as a whole until the last year.

Another pursues the opposite course. He begins with a study of the anatomy as a complete system. He considers its functions, and then goes on to study the organs whose actions make function, and finally to the ultimate elements of which organs and tissues are composed, and whose aberrant functions afford the pathological disturbances with which it is to be his life's work to battle.

The student who spends his first year in a school that begins with histology, and who goes to one that ends its course with tissue elements, never gets beyond elementary matters in his entire college training. This certainly will not tend to make the best practitioners, or to raise our profession to its highest point of efficiency. There should be a comprehension of the benefits of each method, a careful discussion of the merits of all systems of teaching, and an intelligent and discriminating adoption of that which is best. To this end I have accepted the invitation of the executive committee to bring this subject before you.

I am a believer in the analytical system. I think it is easier to arrive at an understanding by taking in pieces that which we do not construct, and thus get at a knowledge of the mysteries of that which we must attempt to repair. Let me give you my reasons for this faith, and then please allow me to listen while you show me wherein I am wrong, or confirm my prepossessions by your own corroborative testimony. Do not then understand me as speaking dogmatically when I propose the following methods in teaching anatomy, but only as offering suggestions.

Our sole reason for examining tissues and organs is that we may learn their action and function. Hence, we should begin with function. This requires that the preliminary examination should be of the system, and not of its organs. The study of anatomy, then, should commence with a general examination of the body as a whole. In a dental school the first year should be devoted to general anatomy, beginning with osteology, or the framework. Then the viscera should be taken up, and their general morphology and function should be studied. This should be followed by myology, syndesmology, and neurology, that a fair idea of the whole body may be claimed. Practical anatomy should be commenced this term, and one extremity dissected. It has sometimes been urged that the student should not dissect until he has learned something of anatomy. This argument would be cogent if the object were to learn how to dissect. But we dissect to learn anatomy, and do not learn anatomy to discover how best to dissect.

All the study of this year should be general. Not a hint of any specialty should be given, and hence the teacher for this year is preferably a medical man. If he is a dentist, he is apt to introduce his specialty too early. The general study of the human body should be finished in the freshman year.

In the second, or junior year, the student begins to differentiate in his study. He should now take up regional anatomy. He has furnished the study of the body as a whole. Not that he has learned all that he should, but he has devoted all the time that can be spared out of a three years' course, and he takes up the study of the part to which he is to devote his attention as a specialist. His field is bounded below by the clavicle, and he must have a special, definite, intimate knowledge of all above that.

As a part of this he commences the study of dental anatomy. The first step in this is comparative dental anatomy,—that is, the study of the dental organs as a whole, precisely as he began the first year in general anatomy. The dentist who learns nothing of the general relations of the teeth, and whose comprehension of them is only that they are organs out of which he is to pick his living, cannot claim any scientific knowledge. The teeth in all the different classes of animals should be generally studied, until the dentition of man is reached, when his teeth should be intimately studied in all their anatomical relations. The anatomy of the second or junior year is, as a whole, devoted to organs, as is that of the first year to systems.

No man can finish the anatomical studies necessary to dental practice in two years. He imperatively needs the third year, and this should be given up to careful examination and investigation of tissues. In this year the microscope is a necessary adjunct. The student has now learned enough of function to comprehend how it modifies, or is modified, by structural development. In this third and finishing year he does not entirely confine his attention to histological anatomy, but he continues regional anatomy, because he isnot yet sufficiently familiar with the organs, especially ofthe head. He also bestows considerable attention upon surgical, and morbid, or pathological anatomy, and he thus finishes his course by attention to the minutiæ and detail for which he is unprepared during his first or second year, because he has not then the general knowledge to allow him fully to comprehend it, and because his mind usually is not sufficiently trained and disciplined to give him mastery over his attention.

The student who thus advances by regular gradations each year, separately taking up and mastering a definite branch or part of the subject, will be likely to retain his knowledge, because he has advanced toward it by a direct route, and because each division is made subsidiary to the next, and there is a regular gradation and progress.

If such a syetem, or if some other regular system, can be adopted in its general features by all of our schools, the grading of one who for any cause changes his college during his course will be greatly facilitated, and he will not be likely to miss any of the subdivisions. Our graduates will be better qualified for practice, and the tone of the profession will be elevated.

I would pursue the same general plan in the study of chemistry and physiology, the other basal studies of the theoretical curriculum. They should extend through the entire course, the last year in each to be devoted to special instructions adapted to an exclusive dental practice.

Materia medica should begin with the first year, but therapeutics cannot be profitably commenced until the student has obtained some knowledge of drugs, and hence it becomes a second- and third-year study, materia medica extending over the first two years.

Embryology properly belongs to the second year, because its study demands an acquaintance with technical terms that are all unfamiliar at the outset, and because it is an intricate and involved matter which requires a disciplined attention. Aside from these, there is no reason why it might not be begun with the freshman year.

Metallurgy is a second-year study, because its consideration demands a good acquaintance with general chemical laws, and these are acquired during the first year.

Surgery is a third-year study, because it demands not only a complete knowledge of anatomy, but a trained hand and absorbed attention as well. The student should begin the study of surgical pathology in the second year, and it may perhaps form a part of his general pathological studies.

Pathology should be differentiated from operative dentistry. They have very little in common, save that each may be curative. But operative dentistry is wholly mechanical and manipulative, while pathology should cover all medicinal and general treatment. Operative dentistry is largely prophylactic, while pathology is so to but a slight degree. Whatever has to do with the action of drugs, whether generally or topically applied, belongs to pathological practice. In the treatment of alveolar abscess, for instance, operative dentistry has very little part, its practice being confined to that which is mechanical, or that which is done with instruments. I believe that in the past we have not sufficiently distinguished between the two. A sharp line of demarkation should be drawn between that which is mechanical and that which is therapeutical.

It will be seen that I have not attempted to assign any place to the practical part of dentistry. My subject was the teaching of anatomy, but I have thought it not inappropriate to suggest some thought concerning other didactic studies.

Let me repeat that I have only considered the matter tentatively, and realize as fully as any of you that there is room for much consideration and extended discussion before the various studies in our curriculum shall each have been definitely assigned its appropriate place.—By Courtesy of F. L. Hise, of *Dental Cosmos.*

